# Modulating Effects of L-Arginine and *Tribulus terrestris* Extract on Fipronil-Induced Interference in the Male Reproductive System of Rats: Antioxidant Potential, Androgen Receptors, and Nitric Oxide Synthase Interplay

**DOI:** 10.3390/toxics13050371

**Published:** 2025-05-02

**Authors:** Doaa H. Elsayed, Ahmed A. Bakhashwain, Eman A. Ahmed, Hatim A. Al-Abbadi, Heba M. A. Abdelrazek, Menna Allah I. El-Menyawy, Wafaa K. Teleb, Noran M. Tawfik, Ibrahim E. Helal, Heba N. Gad EL-Hak

**Affiliations:** 1 Department of Theriogenology, Faculty of Veterinary Medicine, Suez Canal University, Ismailia 41522, Egypt; doaa_hosny@vet.suez.edu.eg; 2 Department of Agriculture, Faculty of Environment Sciences, King Abdulaziz University, Jeddah 80269, Saudi Arabia; aabakhashwain@kau.edu.sa (A.A.B.); ibrahim_hilal@vet.suez.edu.eg (I.E.H.); 3 Department of Pharmacology, Faculty of Veterinary Medicine, Suez Canal University, Ismailia 41522, Egypt; eman_ahmed@vet.suez.edu.eg; 4 Faculty of Medicine, University Hospital, King Abdulaziz University, Jeddah 80269, Saudi Arabia; hatimalabbadi@yahoo.com; 5 Department of Physiology, Faculty of Veterinary Medicine, Suez Canal University, Ismailia 41522, Egypt; 6 Department of Physiology, Faculty of Medicine, Suez Canal University, Ismailia 41522, Egypt; mennaelmenyawi@med.suez.edu.eg; 7 Department of Zoology, Faculty of Science, Suez University, Suez 43518, Egypt; wafaa.teleb@sci.suezuni.edu.eg; 8 Department of Zoology, Faculty of Science, Suez Canal University, Ismailia 41522, Egypt; noran_tawfeek@science.suez.edu.eg; 9 Department of Surgery, Anesthesiology and Radiology, Faculty of Veterinary Medicine, Suez Canal University, Ismailia 41522, Egypt

**Keywords:** androgen receptors, antioxidants, fipronil, male, sex organs, nitric oxide synthase

## Abstract

The protective potentials of *Tribulus terrestris* (TT) and L-arginine (L-Arg) against reproductive toxicity induced by fipronil (FPN) in male rats were investigated. A total of 36 male rats were allocated into six groups: control, TT, L-Arg, FPN, FPN + TT, and FPN + L-Arg groups. The body and sex organ weights, semen criteria, serum testosterone levels, and testicular oxidative stress were determined. Sexual behavior, testicular and penile androgen receptor (AR), penile nitric oxide synthase (NOS), immunohistochemistry of proliferating cell nuclear antigen (PCNA), and histopathology were also assessed. FPN disrupted reproductive health by influencing the expression and activity of NOS and AR, leading to compromised erectile function, sexual dysfunction, and hormonal imbalance. Significant improvements in body weight, reproductive organ weights, the expression of NOS and AR, and testosterone levels were observed in the TT- and L-Arg-treated groups. Behavioral assessments indicated improved sexual performance in the TT- and L-Arg-treated groups. Histopathological studies of the testes and penis tissue, immunohistochemical expression of PCNA in testicular tissues, and biochemical analyses further confirmed the protective effects of TT and L-Arg. Collectively, these findings highlighted the potential of TT and L-Arg in counteracting FPN-induced reproductive impairments.

## 1. Introduction

Pesticides are used in agriculture in developed countries to optimize crop yields [1]. Conversely, these chemicals adversely affect the environment, food safety, and animal and human health [2]. Fipronil (FPN) is a widely utilized N-phenylpyrazole pesticide in veterinary medicine and agriculture [3]. Even though FPN is categorized as a class II hazardous pesticide according to the World Health Organization, it has been associated with retrogressive environmental and health effects [4]. FPN hinders the function of insect γ-aminobutyric acid (GABA)-gated chloride channels [5], which interfere with the neural influx and cause neural excitation, paralysis, and death of the insect [6].

The male reproductive system is notably vulnerable to ecological toxins, including pesticides like FPN [7]. However, increasing concerns have emerged regarding its potential adverse influences on the environment and human health, particularly its impact on reproductive systems. Studies demonstrated that FPN has been associated with a spectrum of reproductive dysfunctions comprising diminished testosterone levels, compromised spermatogenesis, and compromised erectile function [8,9,10]. The underlying mechanisms involving oxidative stress arise from a disparity between the antioxidant response and reactive oxygen species (ROS) generation. This oxidative stress can damage reproductive tissues, disrupt nitric oxide synthase (NOS) activity, and interfere with androgen receptor (AR) signaling, further exacerbating reproductive impairments [11].

Given these concerns, there is an urgent need to explore potential agents that could alleviate the injurious effects of FPN on male reproductive health. Therapeutic agents such as L-arginine (L-Arg) amino acid and *Tribulus terrestris* (TT) have gained attention for their potential protective effects. L-Arg is a vital amino acid in many biological and metabolic body functions [12]. It is a substantial substrate of various enzyme pathways implicated in cellular immunity and cell growth [13,14]. Its deficiency disturbs sperm metabolism, reduces motility, impairs spermatogenesis, and increases sperm abnormalities [12,15]. Furthermore, administration of L-Arg to patients with oligospermic asthenospermic cases enhanced sperm quality regarding motility, vitality, and concentration [16]. Additionally, L-Arg exhibits dramatic effects by accelerating the glycolysis rate, followed by generating ATP, which is fundamental for sperm functions [15]. Moreover, L-Arg can reinforce nitric oxide (NO) production, which is mediated by NOS [17]. NO is a crucial modulator in sperm capacitation via the increment of tyrosine phosphorylation in sperm protein [18]. L-Arg is critical in promoting vascular health and enhancing blood flow, which are essential for erectile function [19].

Botanical treatments have therapeutic indications for their antioxidant ability [20]. *Tribulus terrestris* (TT), the *Zygophyllaceae* family, is used in folk medicine to energize physical fitness and sexual patterns [21]. It has many pharmacological efficacies. TT consists of pivotal bioactive ingredients, especially steroidal saponins, flavonoids, tannins, and alkaloids, that play fundamental roles in its ameliorative pharmacological actions [22]. These actions include antioxidant [23], anti-inflammatory [24], and antimicrobial [25] actions, and augmentation of hormones as well as gonadotropins [23]. TT also has an integral aphrodisiac effect via its androgen-promoting ability [26]. In addition, its capability to improve NO synthesis acts as a signal transduction mediator, enabling relaxation and dilatation of blood vessels, thus strengthening the circulation of the tissues [27]. Hence, TT is beneficial in the treatment of mild cases of erectile dysfunction [28]. Therefore, this research demonstrates the modulating properties of L-Arg and TT on FPN-induced male reproductive toxicity.

This study is the first to examine how L-Arg and TT extract can synergize their effects to modulate FPN-induced male reproductive alterations. This was achieved by investigating sex organ weights and function via the assessment of sperm criteria, blood testosterone levels, testicular androgen receptors (AR) expression, and sexual behavior. Additionally, oxidative stress and penile NOS expression were assayed.

## 2. Materials and Methods

### 2.1. Tribulus Terrestris Extract Preparation

#### 2.1.1. Plant Extraction

*Tribulus terrestris* seeds were obtained from a commercial herbarium, and the authentication process relied on the analysis of morphology and taxonomic elements conducted by the Botany Department at Suez Canal University. The plant was registered with voucher No. 0331. A total of 10 g of TT powder was quantified and subjected to extraction using a 100 mL mixture of methanol and water in an 8:2 ratio. The extraction process involved sonication for 30 min [29]. After centrifuging the sample for 15 min at 12,000 rpm and 4 °C, a 0.22 µm syringe-driven filter was used to filter the supernatant. The filtrate was subjected to concentration at 35 °C with a rotary evaporator [30]. The resulting solid dry extract was stored at −20 °C until needed. To create a stock solution, 50 mg of the lyophilized extract was dissolved in 1 mL of a solvent mixture consisting of acetonitrile, methanol, and water (1:1:2). Complete solubility was attained by vertexing the sample and ultrasonically treating it for 10 min at 30 kHz. The stock solution was diluted to obtain a 1 µg/µL concentration, followed by centrifugation. A 10 µL aliquot was used for LC-MS/MS injection. The analysis was conducted in negative and positive modes, including blank and quality control samples, to ensure experimental reliability [31].

#### 2.1.2. LC-MS/MS Analysis for Compound Characterization

1. Instruments and Acquisition Method

An ExionLC system (AB Sciex, Framingham, MA, USA) equipped with an autosampler was used to separate small molecules, with an in-line filter disk pre-column (0.5 µm × 3.0 mm, Phenomenex, Torrance, CA, USA) and an Xbridge C18 column (3.5 µm, 2.1 × 50 mm) from Waters Corporation (Milford, MA, USA) maintained at 40 °C. The flow rate was set to 300 μL/min. The mobile phase for the positive mode consisted of solution A, 5 mM ammonium formate in 1% methanol with the pH adjusted to 3.0 using formic acid, and solution B, 100% acetonitrile. For the negative mode, solution A comprised 5 mM ammonium formate in 1% methanol with the pH adjusted to 8 using sodium hydroxide. The gradient elution was programmed as follows: 0–5 min, 10% B; 5–20 min, 10% to 90% B; 21–25 min, 90% B; and 25.01–28 min, 10% B, followed by column equilibration at 10% B [32].

Mass spectrometry was carried out on a Triple TOF 5600+ system equipped with a Duo-Spray source in ESI mode (AB SCIEX, Concord, ON, Canada). The IDA method allowed for the simultaneous collection of full-scan MS/MS and MS data, consisting of high-resolution survey spectra ranging from 50 to 1100 *m*/*z*, with a 50 ms survey scan followed by the selection of the top 15 most intense ions for acquiring MS/MS fragmentation spectra [33].

2. LC-MS Data Processing

The open-source MS-DIAL programmer (Version 4.90) [34] analyzed small compounds in the sample in-depth and non-targeted. To confirm features (peaks) from the total ion chromatogram (TIC), criteria including aligned features exhibiting a signal-to-noise ratio greater than 5, and sample-to-blank intensity ratios exceeding 5 were applied. Subsequently, the MS-DIAL output was further processed using PeakView (Version 2.2) in conjunction with the MasterView (Version 1.1) package (AB SCIEX).

### 2.2. Animals

Thirty-six adults male Wistar rats (190 to 200 g) were obtained from the Laboratory Animal House, Faculty of Veterinary Medicine, Suez Canal University, Egypt. Rats were subjected to an adaptation period for 1 week. Rats were caged at 25 ± 2 °C with sawdust covering the floor. Animals were fed a basal diet [35] and water, which were supplied *ad libitum*. The committee of Scientific Research and Biological Ethics for animals used in laboratory experiments in the Faculty of Science, Suez Canal University, Egypt, agreed to all the procedures in the current experiment (No. REC331/2024).

### 2.3. Experimental Design

The experimental rats were categorized into six groups. Group I (control) received daily oral gavage of distilled water. Group II (TT group) rats were given 100 mg/kg 25%w/v TT extract in distilled water, according to Aldaddou, Aljohani [36]. Group III (L-Arg group) rats were given 2% *w*/*v* of 10 mg/kg L-Arg [37]. L-Arg was obtained from NOW Foods (NOW Foods Inc., Bloomingdale, MI, USA; Product Code: 0022-2012-05; Lot Number: 2234567A). The L-Arg was pharmaceutical grade with ≥99.5% purity, as confirmed by HPLC analysis (Certificate of Analysis No. ARG-2023-088). The amino acid was supplied as L-Arg hydrochloride in powder form with a molecular weight of 210.7 g/mol and met USP standards for heavy metal content (<0.001%) and residual solvents (Class 1–3 solvents below ICH Q3C limits). Before administration, L-Arg was freshly dissolved in double-distilled water (pH 7.0) to achieve the required concentration for oral gavage. The solution was prepared daily and administered within 30 min of preparation. Identity and concentration were verified through ninhydrin reaction and spectrophotometric analysis at 570 nm before each preparation batch. In Group IV (FPN group), rats were given 4.85 mg/kg (1/20 LD_50_) FPN [3]. FPN (5-amino-1-[2,6-dichloro-4-(trifluoromethyl) phenyl]-4-[(trifluoromethyl)sulfinyl]-1H-pyrazole-3-carbonitrile) was obtained from StarChem Industrial Chemicals (Catalog No. SC-FPN-250, Wellford, SC, USA). The technical grade FPN had a certified purity of 96.2%, as determined by gas chromatography–mass spectrometry (GC–MS) analysis (Certificate of Analysis No. FPN-2023-04–107; Lot No. FP22030517). The compound was supplied as an off-white crystalline powder with a molecular weight of 437.15 g/mol and CAS Number 120068-37-3. Prior to experimental use, purity was independently verified using HPLC analysis. The FPN was stored in its original sealed container at 2–8 °C in a secured, ventilated cabinet designated for pesticide storage according to institutional chemical safety guidelines. Group V (FPN + TT group) rats were given 1/20 of FPN LD^50^ (4.85 mg/kg FPN) and 100 mg/kg TT extract. Group VI (FPN + L-Arg group) rats were given 4.85 mg/kg FPN (1/20 of FPN LD^50^) and 10 mg/kg L-Arg orally. The different treatments were administered daily via a gastric tube for 63 days.

### 2.4. Body Weights

Body weights were recorded at the experiment’s commencement and termination for each rat.

### 2.5. Male Rat Sexual Behavioral Test

The sexual behavior test was conducted on the 60th day of the experiment. The testing apparatus was composed of a transparent cubic plexiglass box (40 cm × 60 cm × 30 cm) (width × length × height). The floor was covered with 2 cm-thick bedding material so the rats were comfortable during behavior expression. Visual communication between test subjects was prohibited by separating each apparatus from the neighboring one with a cardboard sheet.

Rats were relocated from their cages to the testing apparatus by an accustomed person. Each male rat was positioned and habituated in a plexiglass copulatory arena cage for 5 min. After that, a sexually receptive estrus female rat was presented into the cage and allocated to the testing apparatus for 10 min. A total of six rats/group were tested. The color and light intensity in the testing apparatus were the same as in the home cages for rats. The following male sexual parameters were documented or calculated for the observation period. (1) Latency to 1st behavior: time from introducing the female into the testing arena until the first behavior—i.e., courtship, mount, or intromission. (2) Mount latency: time from introducing the female until the first mount. (3) Mount duration: total duration of mounting behavior over 10 min (4) Mount frequency: the number of mounts in a series. (5) Intromission frequency: the number of intromissions in a series. (6) Copulatory efficiency = (numbers of intromissions/number of mounts) × 100. All the previous behavioral elements were according to Baum [38] and Huijgens, Guarraci [39].

The testing apparatus included an HD video camera (NO. B80, Yes-original.co, Shanghai, China) for video recording and grading of behaviors. A digital video recording device (model No. OR 4CH 5IN1, Yes-original, Shanghai, China) was connected to the camera. Each testing session was analyzed via Behavioral Observation Research Interactive Software (BORIS, Version 2.95, University of Torino, Torino, Italy). After the 10 min testing session, the apparatus was cleared of fecal droplets and thoroughly wiped with 40% ethyl alcohol, and the bedding materials were changed to avoid the transmission of odor between subjects.

### 2.6. Sample Collection

After the end of the 63rd day of the experiment duration, retro-orbital blood samples were obtained under light tetrahydrofuran anesthesia. The obtained sera were stored at −80 °C until they were analyzed. Animals were sacrificed and dissected. Genital organs, including the testicles, the tail of the epididymis, the prostate gland, and the seminal glands, were dissected and weighed. By dissecting the tail of the epididymis, epididymal sperm were analyzed for sperm motility, viability, abnormalities, and count, as described by Aldaddou, Aljohani [36]. For motility assessment, 10 μL of sperm suspension was examined under light microscopy (400× magnification), with 200 spermatozoa counted in multiple fields and classified into progressive motility, non-progressive motility, and immobility. Throughout the analysis, the temperature was consistently maintained at 37 °C to preserve sperm viability. The eosin–nigrosine exclusion method was employed to determine sperm viability; wherein viable spermatozoa exclude the stain while non-viable cells exhibit pink coloration. Morphological evaluation was performed under oil immersion (1000× magnification) by analyzing 200 spermatozoa for structural abnormalities in the head, mid-piece, and tail regions, with results expressed as percentages. The caudal epididymis was meticulously excised and submerged in 2 mL of physiological saline maintained at 37 °C. Small incisions were created to facilitate sperm dispersal during a 10–15 min incubation period. Sperm concentration was quantified using an improved Neubauer hemocytometer following a 1:20 dilution with formal saline, and calculations were based on counts from five standard squares. Testes were weighed and homogenized in cold neutral phosphate buffer and then centrifuged in a cold centrifuge at 4000 rpm. The harvested supernatants were stored at −80 °C till the analysis for oxidants and antioxidant markers.

### 2.7. Hormonal and Biochemical Analysis

Serum testosterone hormone was measured with the ELISA technique using diagnostic commercial kits for testosterone (Bioassay Technology Laboratory Co., Shanghai, China). The testicular oxidant and antioxidant markers were determined using rat commercial kits, including superoxide dismutase (SOD), catalase activity, total antioxidant capacity (TAC), and malondialdehyde (MDA), as indicators for lipid peroxidation with Cat No. MBS266897, MBS726781, MBS9718973, and MBS2540407, respectively (MyBioSource Co., San Diego, CA, USA).

### 2.8. Histopathology

Testicular and penile tissues were subjected to fixation in 10% neutral buffered formalin purchased from Thermo Fisher Scientific (Catalog No. SF100-4, Waltham, MA, USA). The fixative solution contained 10% (*v*/*v*) formaldehyde (4% formaldehyde by weight) in phosphate-buffered saline with a pH of 7.2–7.4 at 25 °C and was used immediately after sacrifice to preserve the morphology and prepare for paraffin sections. With the aid of a microtome, cross sections of the fixed testicular tissue (5 μm thick) were sectioned. The testicular and penile sections were stained using hematoxylin and eosin staining (H&E), which was performed using Harris Hematoxylin (Catalog No. HHS32, Sigma-Aldrich, St. Louis, MO, USA) and Eosin Y Solution (Catalog No. HT110332, Sigma-Aldrich, St. Louis, MO, USA) [40]. Moreover, the penile and testicular sections were stained with Masson’s Trichrome stain [41]. The sections stained with H&E were observed under a light microscope to evaluate histological characteristics, including cell organization and any signs of damage. Ten seminiferous tubules per testis were evaluated for spermatogenesis (Johnson testicular score), according to Johnson et al. [42].

Five sections and five fields of each testis were photographed from the penile and testicular sections stained with Masson’s Trichrome stain. The total collagen area was calculated using ImageJ software (Version 1.54). For the histomorphometry of seminiferous tubules, 10 seminiferous tubules per testis were used to determine the mean tubular diameter, and the height of spermatogenesis was measured using ImageJ.

### 2.9. Immunohistochemistry for PCNA Detection

Testicular and penile paraffin-fixed testicular sections (5 μm thick) were subjected to positively charged slides for proliferating cell nuclear antigen (PCNA) immunohistochemistry using rat monoclonal anti-PCNA primary antibody (ab92552, Abcam, Cambridge CB2 0AX, UK) at a dilution of 1:100 in phosphate-buffered saline. Procedures for IHC and its quantitative analysis were carried out as described by El-Sakhawy, Abusaida [43]. The number of immunohistochemical PCNA-positive cells was quantified. Five sections and five slides of each testis were photographed and evaluated.

### 2.10. Quantitative Real-Time PCR

Total RNA was isolated from testicular and penile tissues using the ABT Total RNA mini extraction kit (Applied Biotechnology, Cat. No. ABT002, Cairo, Egypt) following the manufacturer’s instructions. The extracted mRNA was reverse transcribed to cDNA using the cDNA synthesis ABT 2x RT Mix Oligo kit (Applied Biotechnology, Cat. No. AMP11, Cairo, Egypt) according to the manufacturer’s protocol. A quantitative real-time polymerase chain reaction (qRT-PCR) was carried out to estimate AR and NOS expression levels using the Applied Biosystems StepOne™ Real-Time PCR System. The reaction was performed using the ABT 2× qPCR Mix (SYBR) High ROX kit (Cat. No. AMP04, Applied Biotechnology, Ismailia, Egypt). The sets of primer arrangements are shown in Table 1. The conditions for the thermal cycling were 45 cycles at 95 °C for 10 min, 95 °C for 15 s followed by 59 °C for 40 s, and finally 72 °C for 40 s. The attained data underwent normalization using the housekeeping gene β-actin. Finally, the fold expression results were analyzed using the 2^−ΔΔCt^ method [44].

### 2.11. Statistical Analysis

The data were initially evaluated for normality using the Shapiro–Wilk test. Normally distributed data were analyzed using one-way analysis of variance (ANOVA), followed by Tukey’s significant difference test as a post hoc comparison to determine significant differences between groups. Non-normally distributed data were evaluated using the Kruskal–Wallis test. Spearman’s rank correlation was implemented to assess the relationships among the non-normally distributed variables. Principal component analysis (PCA) was performed using the varimax rotation method to reduce the dimensionality of the dataset and identify underlying patterns in the data. All statistical analyses, data visualization, and PCA were conducted using SPSS (Version 29). A *p*-value of <0.05 was considered statistically significant.

## 3. Results

### 3.1. LC-MS/MS Analysis of TT

Thirty-one chemical characteristics of identified compounds of TT found in both negative and positive modes are displayed in Table S1.

### 3.2. Body and Relative Sex Organ Weights

**Figure 1:**
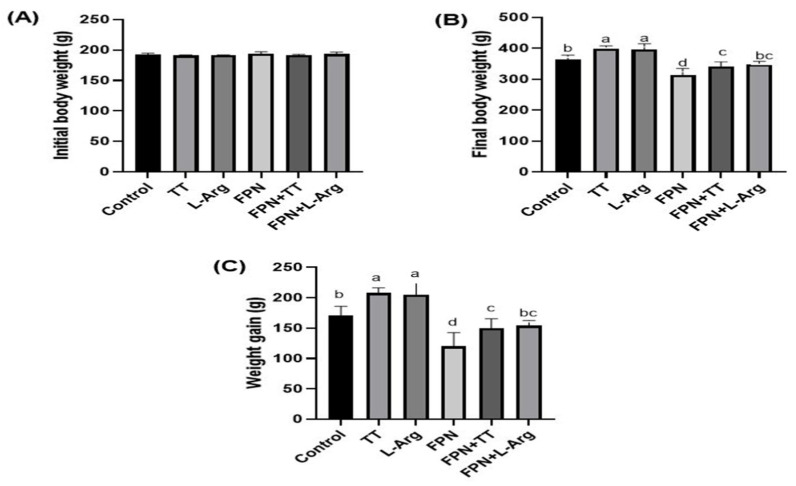
Effect of *Tribulus terrestris* (TT) and L-arginine (L-Arg) on (**A**) initial body weights, (**B**) final body weights and (**C**) weight gain in fipronil-treated male Wistar rats. Different letters indicate significance at *p* ≤ 0.05.

The initial body weight showed non-significant variations between different groups (Figure 1A). Both weight gain (WG) and final body weight (FBW) were statistically (*p* ≤ 0.05) increased in the TT and L-Arg groups compared to the control and other treatment groups (Figure 1B,C). However, FPN-treated rats had the lowest (*p* ≤ 0.05) FBW and WG when matched to other groups. The gavage of TT and L-Arg to FPN-treated rats statistically (*p* ≤ 0.05) improved the FBW and WG equated to the FPN group.

**Figure 2:**
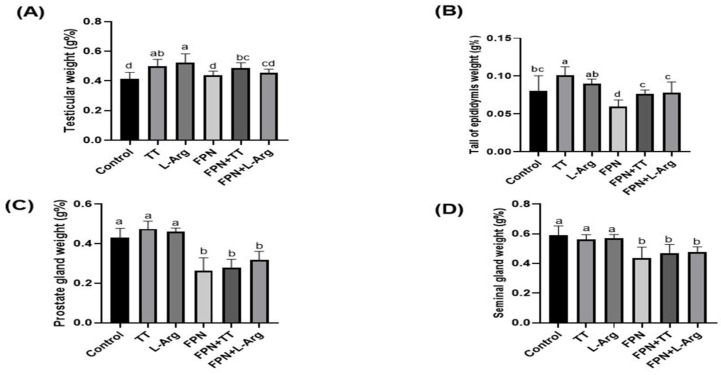
Effect of *Tribulus terrestris* (TT) and L-arginine (L-Arg) on relative sex organ weights (**A**) testicular weight, (**B**) tail of epididymis weight, (**C**) prostate gland weight and (**D**) seminal gland weight of fipronil (FPN)-treated male Wistar rats. Different letters indicate significance at *p* ≤ 0.05.

Regarding the relative weights of testicles and tails of the epididymis, the TT and L-Arg groups exhibited the highest (*p* ≤ 0.05) values in comparison to the other treatment groups (Figure 2A,B). Moreover, the FPN + TT and FPN + L-Arg groups revealed a statistical increase (*p* ≤ 0.05) compared to the FPN-treated rats. The control groups showed a statistical (*p* ≤ 0.05) diminution in relative epididymal weight compared to the TT group. Concerning the relative weights of prostate and seminal glands, the control, TT, and L-Arg groups demonstrated the maximum weights (*p* ≤ 0.05) compared to the FPN-, FPN + TT-, and FPN + L-Arg-treated groups (Figure 2C,D).

### 3.3. Testosterone Hormone and Semen Criteria

**Figure 3:**
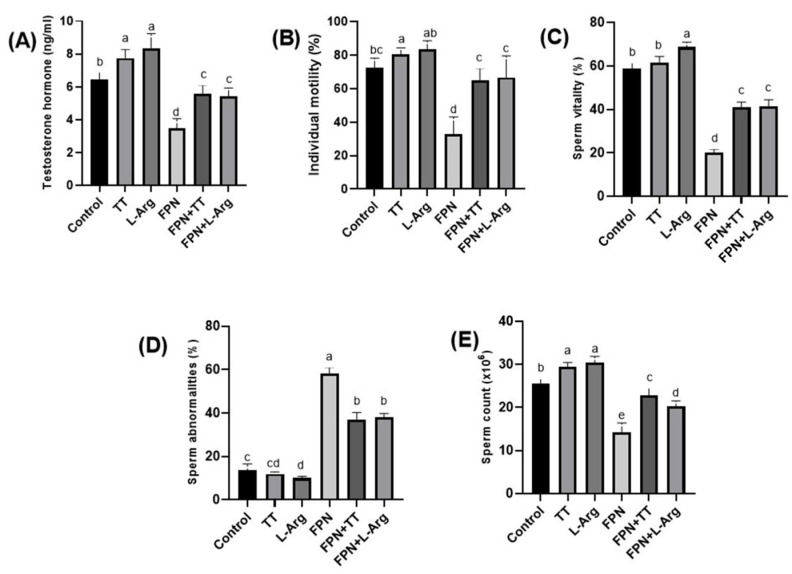
Effect of *Tribulus terrestris* (TT) and L-arginine (L-Arg) on serum testosterone levels and semen criteria in fipronil (FPN)-treated male Wistar rats. Different letters indicate significance at *p* ≤ 0.05.

*Tribulus terrestris-* and L-Arg-treated rats had the highest serum testosterone levels (*p* ≤ 0.05) compared to other groups. The FPN group had the lowest (*p* ≤ 0.05) testosterone levels of the different groups. Conversely, the addition of TT and L-Arg to FPN-treated rats resulted in statistically (*p* ≤ 0.05) significant testosterone elevations compared to the FPN group (Figure 3A).

The progressive forward sperm motility % showed a statistical (*p* ≤ 0.05) improvement in the TT and L-Arg groups compared to the other treated groups. Significant (*p* ≤ 0.05) improvement was noticed in both the FPN + TT and FPN + L-Arg groups regarding progressive sperm motility when compared to the FPN group, which had the lowest (*p* ≤ 0.05) percentage among the groups (Figure 3B).

The L-Arg group demonstrated a statistical (*p* ≤ 0.05) improvement in sperm vitality % compared to other groups. On the other hand, the lowest values of vitality % were recorded in FPN-treated rats (*p* ≤ 0.05). A statistical (*p* ≤ 0.05) amelioration in vitality % was seen in the FPN + TT and FPN + L-Arg groups compared to the FPN group (Figure 3C).

The most significant (*p* ≤ 0.05) increase in sperm abnormalities was seen in FPN-intoxicated rats compared to other groups. However, the least (*p* ≤ 0.05) abnormalities were observed in the TT and L-Arg groups compared to other treated rats. Non-statistical (*p* > 0.05) differences were detected in the abnormality percentages of the control and TT groups (Figure 3D).

The highest (*p* ≤ 0.05) sperm concentrations were obtained in the TT and L-Arg groups compared to the other groups. However, the lowest sperm count values were estimated in the FPN groups compared to the other groups. The sperm counts improved statistically (*p* ≤ 0.05) in the FPN + TT and FPN + L-Arg groups when compared with the FPN group (Figure 3E).

### 3.4. Oxidant and Antioxidant Markers

**Figure 4:**
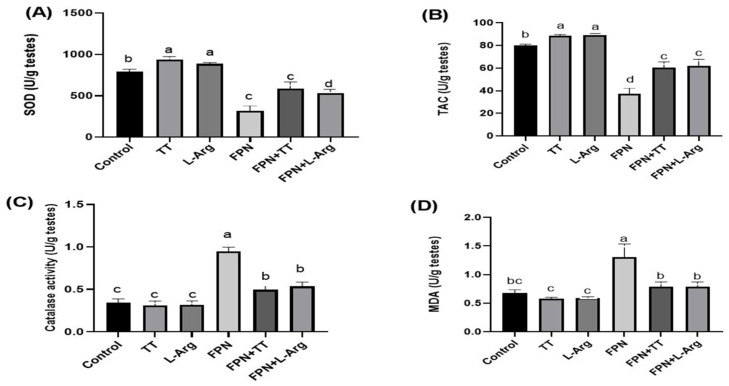
Effect of *Tribulus terrestris* (TT) and L-arginine (L-Arg) on testicular oxidant/antioxidant markers in fipronil (FPN)-treated male Wistar rats. Different letters indicate significance at *p* ≤ 0.05.

The antioxidant markers, SOD and TAC, increased statistically (*p* ≤ 0.05) in TT- and L-Arg-treated rats. Moreover, the FPN + TT and FPN + L-Arg groups revealed a statistically (*p* ≤ 0.05) significant increase in testicular SOD and TAC when compared to FPN-treated rats (Figure 4A,B). The latter group displayed the lowest (*p* ≤ 0.05) antioxidant markers compared to the other groups.

The FPN group demonstrated the highest (*p* ≤ 0.05) catalase activity and MDA testicular values. Nevertheless, the lowest (*p* ≤ 0.05) values were noted in the control, TT, and L-Arg groups. Both the FPN + TT and FPN + L-Arg groups displayed a statistical decrease in testicular MDA levels and catalase activity compared to FPN-treated rats (Figure 4C,D).

### 3.5. Results of Male Rats’ Sexual Behavior in Different Treatment Groups

**Figure 5:**
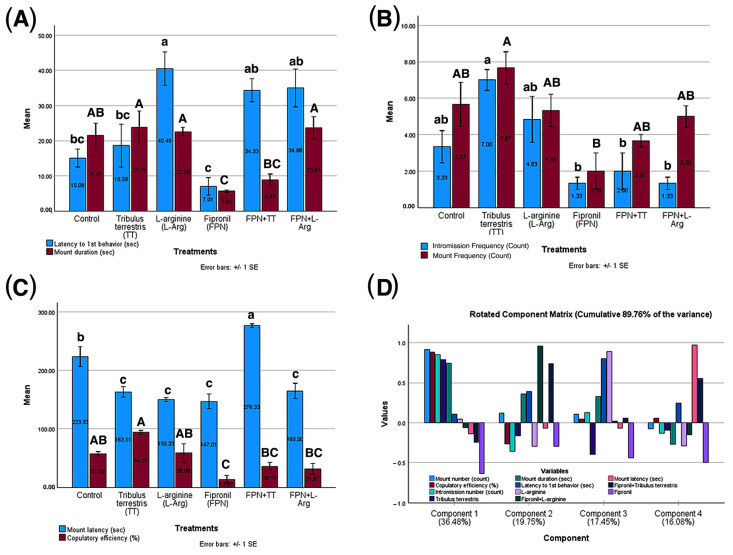
Effect of *Tribulus terrestris* (TT) and L-arginine (L-Arg) on sexual behavior in male Wistar rats. (**A**) Latency to first behavior (sec) and mount duration (sec), (**B**) intromission number (count) and mount number (count), (**C**) mount latency (sec) and copulatory efficiency (%), and (**D**) principal component analysis (PCA). Different letters indicate significance at *p* ≤ 0.05.

A one-way ANOVA revealed significant differences among treatments (control, TT, L-Arg, FPN, FPN + TT, and FPN + L-Arg) on male sexual function metrics (*p* < 0.05). FPN exhibited detrimental impacts, including reduced duration and intromission number and decreased copulatory efficiency (*p* < 0.01). Conversely, TT and L-Arg demonstrated beneficial effects, showing increased latency to first behavior and mount number compared to FPN and improving mount duration, intromission number, and copulatory efficiency (*p* < 0.05). Combination treatments (FPN + TT and FPN + L-Arg) showed enhanced effects compared to FPN alone, suggesting a potential synergistic interaction (*p* < 0.001) and, in the case of FPN + L-Arg, a trend toward increased copulatory efficiency compared to FPN (*p* = 0.08). These findings indicate that TT and L-Arg may counteract the adverse influences of FPN and may help mitigate the negative impacts of FPN on sexual function, with significant implications for the development of therapeutic strategies to address toxin-induced reproductive impairments (Figure 5).

The principal component analysis revealed a multifaceted structure of relationships among the variables across four components, explaining a cumulative 89.76% of the variance. Component 1, accounting for 36.48% of the variance, showed strong positive loadings for TT (0.785), mount duration (0.742), intromission number (0.851), mount number (0.914), and copulatory efficiency (0.885). In contrast, FPN showed a moderate negative loading (−0.638), suggesting a component reflecting overall sexual performance. Component 2, representing 19.75% of the variance, was characterized by strong positive loadings for the combined treatments FPN + TT (0.737) and FPN + L-Arg (0.960) and a weak positive loading for mount duration (0.361). Component 3, explaining 17.45% of the variance, was dominated by a strong positive loading for L-Arg (0.887) and latency to first behavior (0.799). Component 4, accounting for 16.08% of the variance, primarily reflected mount latency (0.968) and showed a moderate negative loading for FPN (−0.499). This analysis highlights that the performance-related variables are controlled mainly by Component 1, with TT positively associated with these metrics and FPN negatively associated. The combined treatments primarily influenced Component 2. Component 3 reflected the relationship between L-Arg and latency to first behavior, and Component 4 was predominantly influenced by mount latency (Figure 5).

### 3.6. Histopathological Observation

**Figure 6:**
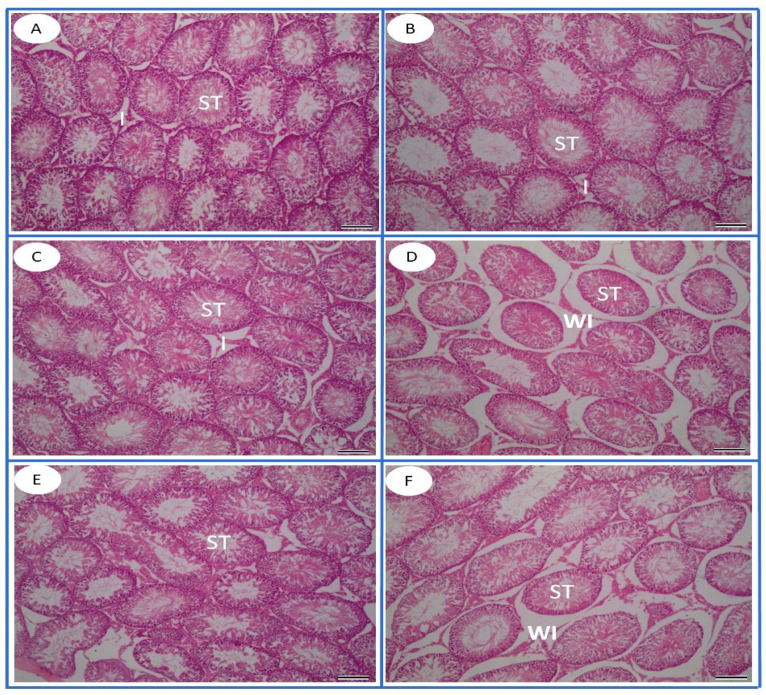
Photomicrograph of H&E-stained testicular tissue sections of the (**A**) control, (**B**) *Tribulus terrestris* (TT), (**C**) L-arginine (L-Arg), (**D**) fipronil (FPN), (**E**) fipronil + *Tribulus terrestris* (FPN + TT), and (**F**) fipronil + L-arginine (FPN + L-Arg) groups. Control, TT, and L-Arg-treated rats revealed regular testicular architecture with multiple closely packed seminiferous tubules (ST) with narrow interstitial (I) spaces in between. The testicular architecture in the FPN group displayed disarrangement and disruption of seminiferous tubules with wide interstitial spaces in between (WI). H&E stain, 100×.

The light microscopy assessment of H&E-stained testicular sections, obtained from the control, TT, and L-Arg groups, revealed regular testicular architecture with multiple closely packed seminiferous tubules with narrow interstitial spaces in between. The testicular tubules were mainly lined by three types of cells: spermatogonia, spermatocytes, and Sertoli cells. The latter exhibited large, pale, vesicular nuclei. Spermatogonia were basally located and exhibited oval to round nuclei. Large round nuclei with various chromatin condensation patterns characterized primary spermatocytes. Early and late spermatids lay in the luminal compartments of the tubules (Figure 6A–C). On the other hand, the testicular architecture in the FPN group displayed numerous degenerative changes, including tubular epithelial vacuolation, focal loss of spermatogenic cells, basal lamina irregularity, edema, and dilation of the blood vessels between the seminiferous tubules (Figure 6D). The FPN + L-Arg group exhibited a marked alleviation and restoration of normal testicular appearance (Figure 6E). However, the FPN + TT group demonstrated tubular epithelial vacuolation. The testicular tissue of this group showed less damage when compared to the FPN group (Figure 6F).

**Figure 7:**
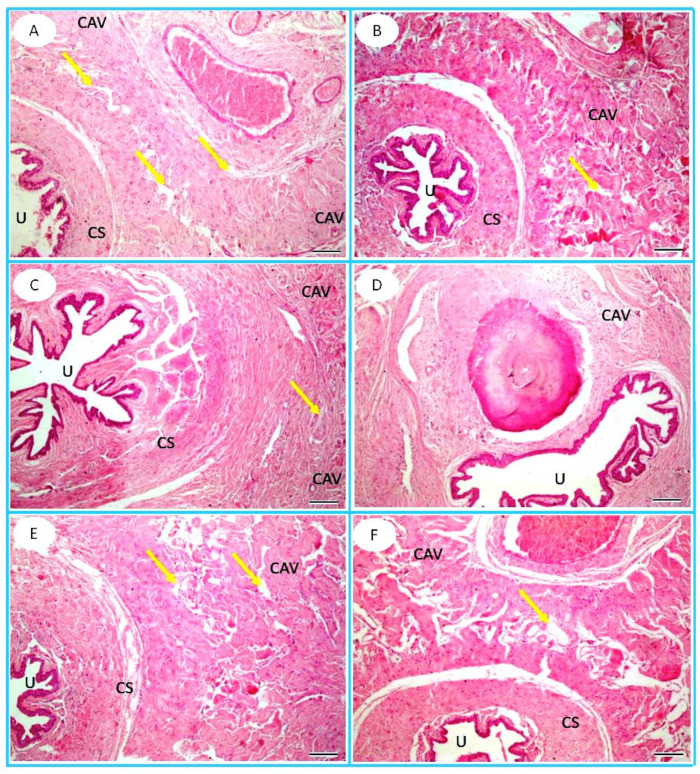
Photomicrograph of cross sections of penile shafts of the (**A**) control, (**B**) *Tribulus terrestris* (TT), (**C**) L-arginine (L-Arg), (**D**) fipronil (FPN), (**E**) fipronil + *Tribulus terrestris* (FPN + TT), and (**F**) fipronil + L-arginine (FPN + L-Arg) groups. Corpora cavernosa (CAV); corpus spongiosum (CS) that surrounds the penile urethra (U); wide, irregular, vascular cavernous spaces (yellow arrows). H&E stain, 100×.

The penile tissue of the FPN-treated group revealed a reduction in cavernous spaces compared to the control group. However, the administration of TT and L-Arg resulted in an improvement in cavernous spaces compared to the FPN group (Figure 7).

**Figure 8:**
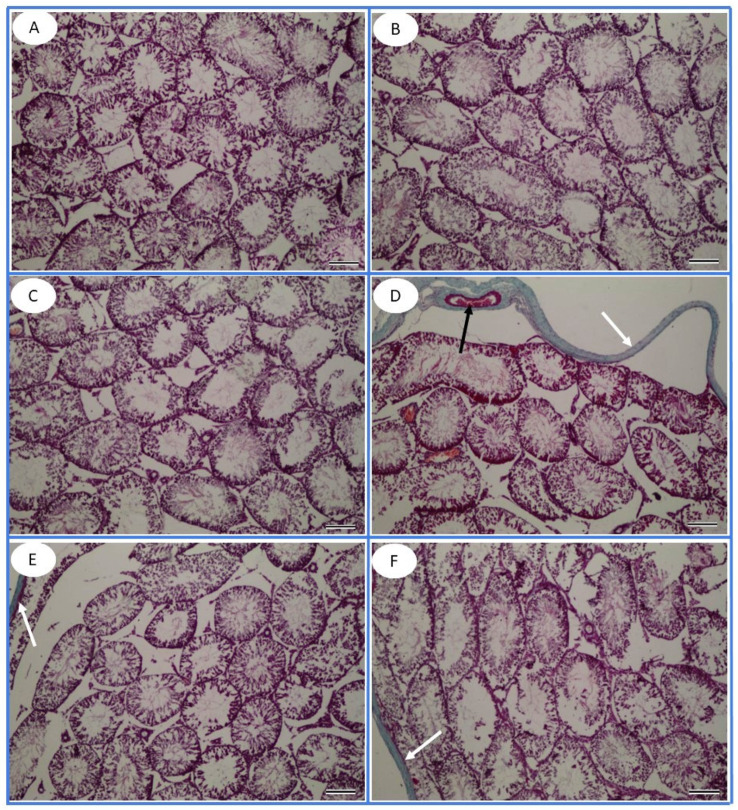
Masson’s trichrome-stained testicular tissue sections of the (**A**) control, (**B**) *Tribulus terrestris* (TT), (**C**) L-arginine (L-Arg), (**D**) fipronil (FPN), (**E**) fipronil + *Tribulus terrestris* (FPN + TT), and (**F**) fipronil + L-arginine (FPN + L-Arg) groups. The collagen fibrous content of the testis was slightly increased in the tunica albuginea (white arrows) of the FPN, FPN + L-Arg, and FPN + TT groups and further decreased in the control, TT, and L-Arg groups. Interestingly, collagen fibers were not recorded within the interstitial spaces of the control and experimental groups. Congested blood vessels appear within the FPN groups (black arrow) (100×).

In Masson’s trichrome-stained testicular sections, the collagen fibrous content of the testis was slightly increased in the tunica albuginea of the FPN, FPN + L-Arg, and FPN + TT groups, respectively *(*Figure 8).

**Figure 9:**
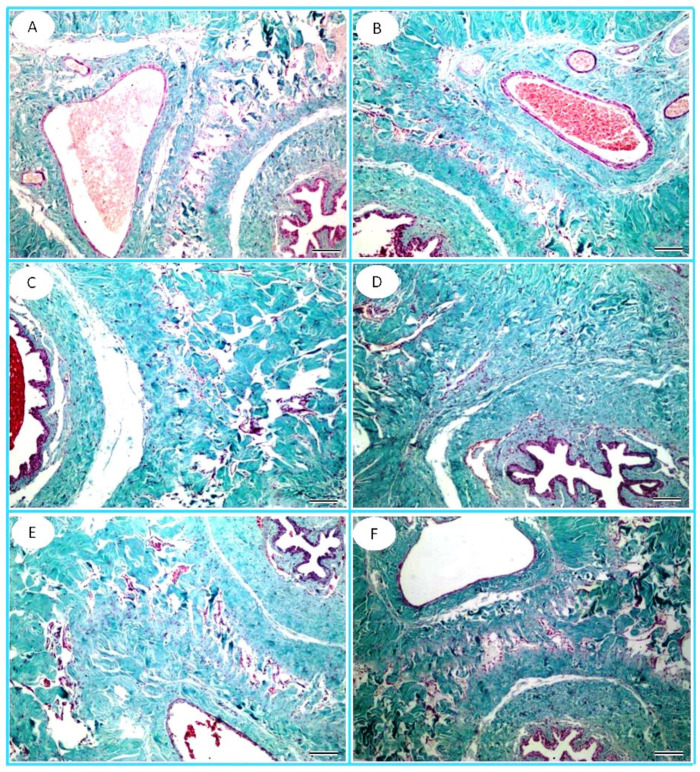
Photomicrograph of cross sections of penile shafts of the (**A**) control, (**B**) *Tribulus terrestris* (TT), (**C**) L-arginine (L-Arg), (**D**) fipronil (FPN), (**E**) fipronil + *Tribulus terrestris* (FPN + TT), and (**F**) fipronil + L-arginine (FPN + L-Arg) groups. The corpora cavernosa of the FPN group revealed marked narrowing of the cavernous spaces, which appeared to have thick bands of collagen fibers. The rats of the FPN + TT and FPN + L-Arg groups exhibited some collagenic fibrous content. Masson’s Trichrome stain (100×).

In Masson’s trichrome-stained penile sections, the corpora cavernosa of the FPN group revealed marked narrowing of the cavernous spaces, which appeared to have thick bands of collagen fibers. The rats in the FPN + TT and FPN + L-Arg groups exhibited low collagenic fibrous contents (Figure 9).

**Figure 10:**
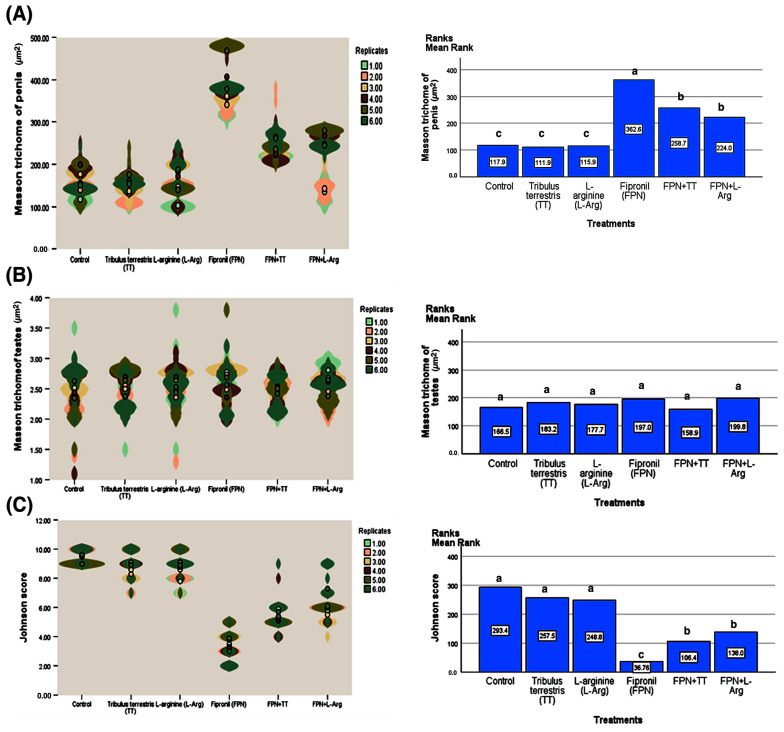
Violin plots, Kruskal–Wallis tests, and Spearman’s rho correlation coefficients for some of the histopathological and spermatogenic parameters across the treatment groups. (**A**) Masson’s trichrome staining of the penis, (**B**) Masson’s trichrome staining of the testes, and (**C**) Johnson score. The Kruskal–Wallis tests revealed significant differences in histopathological and spermatogenic parameters across the treatment groups. Specifically, the Masson’s trichome staining of the penis (χ^2^ = 262.1, df = 5, *p* < 0.001), Masson’s trichrome staining of the testes (χ^2^ = 7.43, df = 5, *p* = 0.190), and Johnson score (χ^2^ = 292.1, df = 5, *p* < 0.001) all showed significant differences in their distributions. Means with different letters are significantly different (*p* < 0.01).

**Figure 11:**
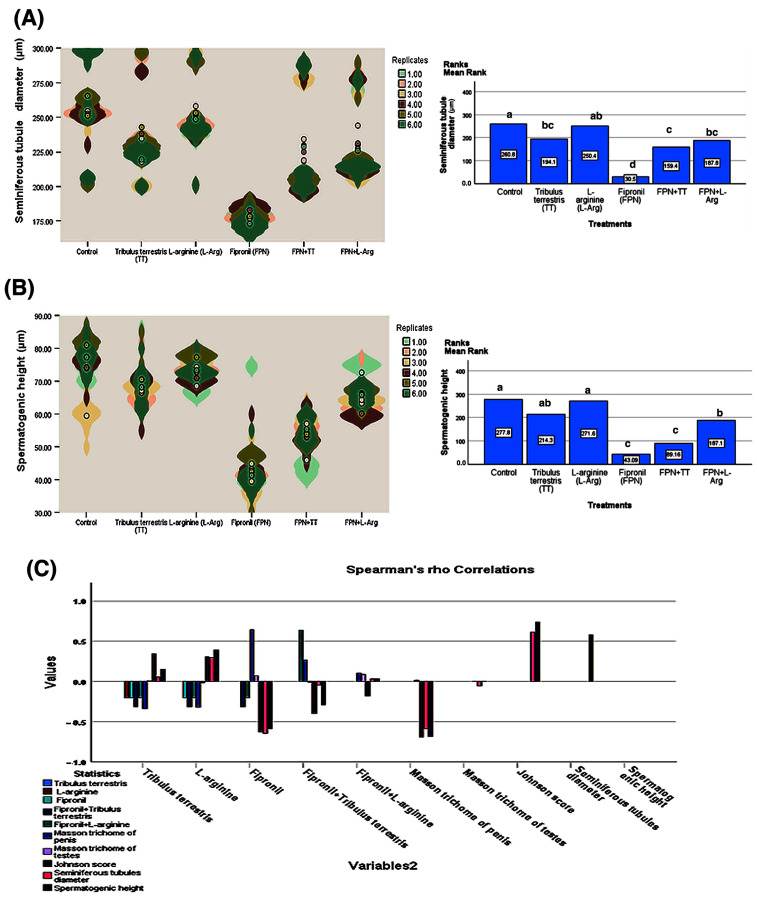
Violin plots, Kruskal–Wallis tests, and Spearman’s rho correlation coefficients for some of the histopathological and spermatogenic parameters across the treatment groups. (**A**) Seminiferous tubule diameter, (**B**) spermatogenic height, and (**C**) Spearman’s rho correlation coefficient. The Kruskal–Wallis tests revealed significant differences in histopathological and spermatogenic parameters across the treatment groups. Specifically, seminiferous tubule diameter (χ^2^ = 191.6, df = 5, *p* < 0.001) and spermatogenic height (χ^2^ = 256.0, df = 5, *p* < 0.001) showed significant differences in their distributions. Means with different letters are significantly different *(p <* 0.01).

Mean rank comparisons, corrected for multiple testing using the Bonferroni correction, further elucidated the specific effects of each treatment on the measured parameters (Figure 10 and Figure 11). Regarding Masson’s trichrome staining of the penis, significant differences were observed across numerous treatment pairs. FPN consistently showed significantly lower mean ranks (362.6) than all other treatments (*p* < 0.001). Likewise, analysis of the Johnson score revealed that the FPN treatment had significantly lower scores (36.76) compared to all other treatments. On the other hand, the treatments did not substantially affect Masson’s trichrome staining of the testes. Only the comparison between FPN + TT and FPN + L-Arg showed a significant difference (*p* = 0.030), as shown in Figure 10. Significant differences were observed between FPN and all other groups (*p* < 0.001). Similarly, the analysis of seminiferous tubule diameters showed that the FPN group displayed significantly smaller diameters (30.50) than all other groups, with *p* < 0.001. Furthermore, in the analysis of spermatogenic height, FPN exhibited a considerably lower mean rank (43.09) compared to the other treatments (*p* < 0.001). FPN showed significant differences compared to the other treatments and FPN + L-Arg, FPN + TT, and TT (Figure 11).

Spearman’s rank-order correlations were conducted to evaluate the associations between treatment groups and the measured histopathological and spermatogenic parameters (Figure 11). The most reported finding was the strongly significant (*p* < 0.01) negative correlation between FPN and multiple indices of spermatogenic health and penile structure. Specifically, FPN exhibited significant negative correlations with the Johnson score (r = −0.63), seminiferous tubule diameter (r = −0.65), and spermatogenic height (r = −0.59) and a positive correlation with Masson’s trichrome staining of the penis (r = 0.64), indicating a detrimental effect on these parameters. Negative correlations between FPN and TT, as well as L-Arg, further supported these findings. Moreover, the Johnson score, seminiferous tubule diameter, and spermatogenic height demonstrated positive intercorrelations, reflecting a coordinated influence on spermatogenic processes. Notably, there were non-significant correlations between the treatments used and Masson’s trichrome staining of the testes. Notably, TT and L-Arg showed positive correlations with several key indicators of spermatogenesis. *Tribulus terrestris* showed a strongly significant (*p* < 0.01) positive correlation with the Johnson score (r = 0.34), and L-Arg correlated with the Johnson score (r = 0.31), seminiferous tubule diameter (r = 0.30), and spermatogenic height (r = 0.39).

### 3.7. Immunohistochemistry

**Figure 12:**
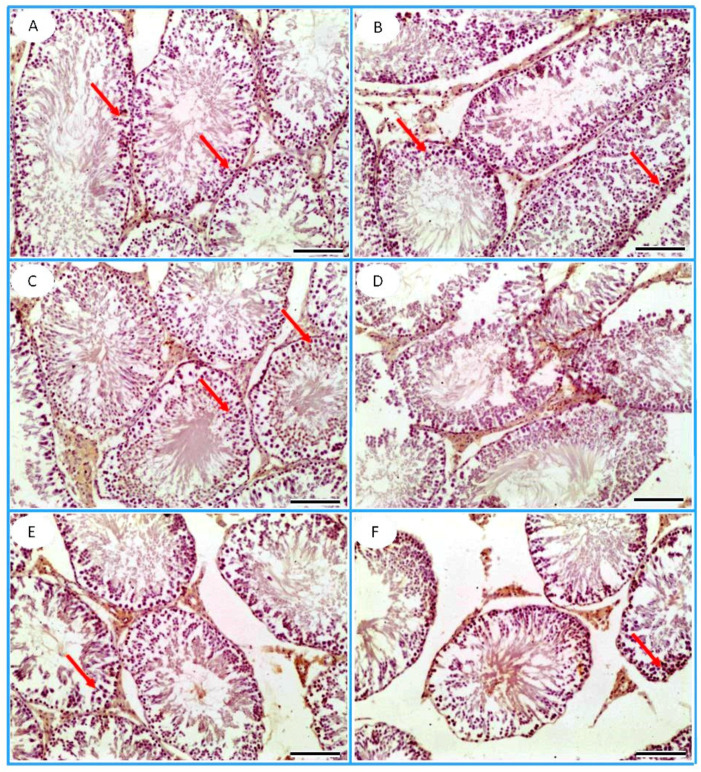
PCNA immunohistochemical staining of testicular tissues of the (**A**) control, (**B**) *Tribulus terrestris* (TT), (**C**) L-arginine (L-Arg), (**D**) fipronil (FPN), (**E**) fipronil + *Tribulus terrestris* (FPN + TT), and (**F**) fipronil + L-arginine (FPN + L-Arg) groups demonstrated many PCNA-immunopositive germ cells (red arrows) within the seminiferous tubules. The FPN group revealed few PCNA-immunopositive germ cells. Meanwhile, many PCNA-immunopositive germ cells were recorded in the seminiferous tubules of the FPN + TT and FPN + L-Arg groups.

The PCNA immunostaining was demonstrated as an intranuclear signal within the spermatogenic cells (Figure 12).

**Figure 13:**
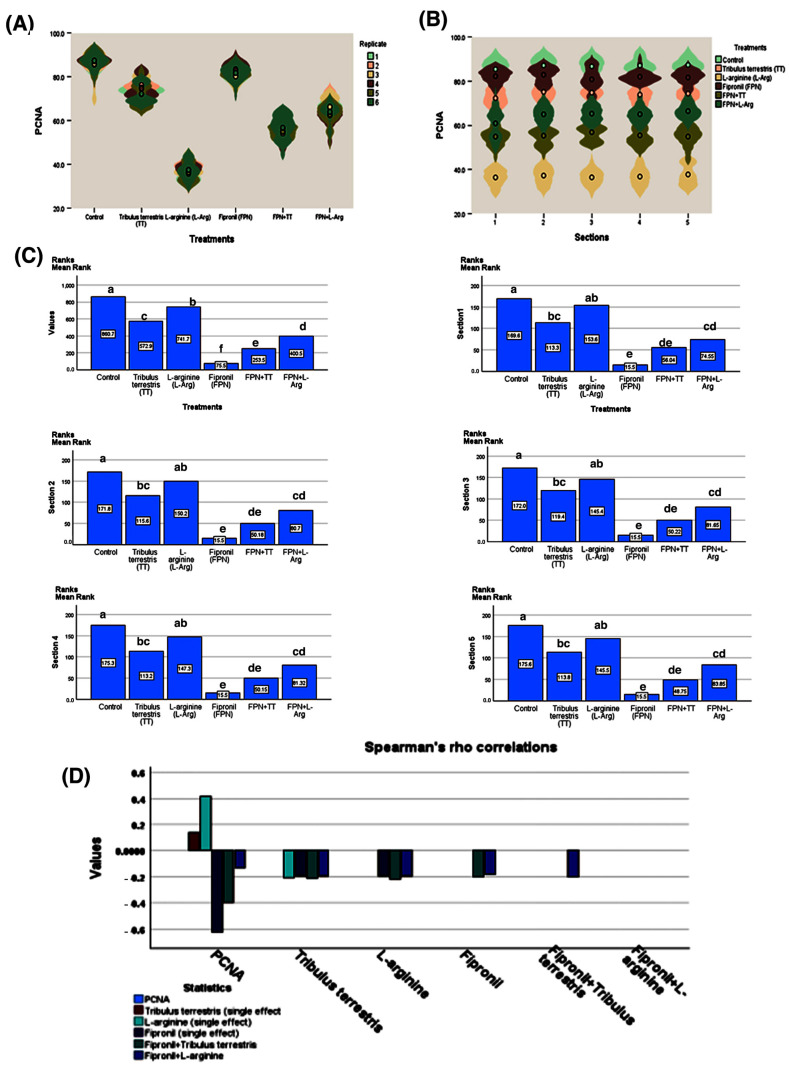
Violin plots, Kruskal–Wallis tests, and Spearman’s rho correlation coefficients of PCNA and spermatogenic parameters among the treatment groups. (**A**) Violin plot of PCNA by replicate, (**B**) violin plot of PCNA by section, (**C**) mean rank comparison of treatments and sections, and (**D**) Spearman’s rho correlation coefficient (*p* < 0.001). Means with different letters are significantly different *(p <* 0.01).

Analysis of the data presented in Figure 13 using the Kruskal–Wallis H test (H = 896.435, *p* < 0.001) revealed substantial differences in PCNA levels across various treatments. The control group demonstrated the highest mean rank (860.66), significantly exceeding all other treatments (*p* < 0.01), particularly FPN (mean rank = 75.50), which showed a pronounced reduction in PCNA levels. On the contrary, TT (mean rank = 572.86) and L-Arg (mean rank = 741.68) displayed elevated ranks, indicating their potential to improve PCNA levels, with L-Arg exhibiting more substantial effects than TT. Mean rank comparisons validated the deleterious effects of FPN while showing that TT and L-Arg partially counteracted these negative impacts, especially in their single applications.

Spearman’s correlation analysis additionally demonstrated a weak positive relationship between PCNA and TT (rho = 0.138, *p* < 0.01) and a more significant positive correlation with L-Arg (rho = 0.414, *p* < 0.01), highlighting their contributions to improved PCNA-associated outcomes. On the other hand, FPN reported a strong negative correlation with PCNA (rho = −0.625, *p* < 0.01), and when combined with TT (rho = −0.396) or L-Arg (rho = −0.131), its adverse impacts diminished their beneficial effects. These findings underline the promising therapeutic potential of TT and L-Arg in mitigating FPN-induced suppression of PCNA activity, drawing attention to their roles in supporting cell proliferation dynamics.

### 3.8. Quantitative Real-Time PCR

**Figure 14:**
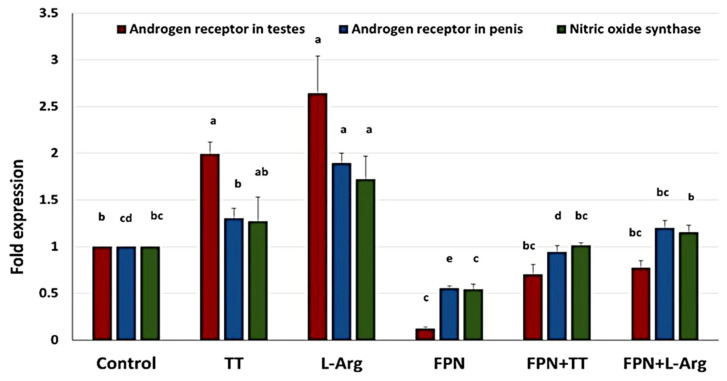
Fold change of testicular androgen receptor (AR), penile AR, and penile nitric oxide synthase (NOS). Data are presented as mean ± SEM and were analyzed using one-way ANOVA, followed by the post hoc Duncan test. Different letters among treatment groups indicate statistical significance at *p* < 0.05.

The transcript levels of androgen and NO were detected using qRT-PCR to evaluate the amelioratory effect of L-Arg and TT against the toxicity of FPN in adult Wistar rats (Figure 14). Our findings revealed that the AR expression level in the testicular tissue was statistically upregulated in the L-Arg and TT groups compared to other groups. Nevertheless, it was statistically downregulated in the FPN group compared to the control group. There was no statistical change in the transcript level of AR in the testicular tissue between the FPN + TT and the FPN + L-Arg groups, which equated to that of the FPN group.

Furthermore, the transcript level of AR in the penile tissue was significantly upregulated in the L-Arg and TT groups compared to the control group. In particular, a statistical decline in the expression level of AR in the penile tissue was detected in the FPN group in comparison to the other groups. Meanwhile, the FPN co-administered with L-Arg and the FPN co-administered with TT showed statistically significant increments in the mRNA expression level of the AR in the penile tissue compared to the FPN group. The NOS mRNA transcript level was also significantly increased in the L-Arg group. However, no significant change was noticed in the transcript level of NOS in the FPN + TT and FPN + L-Arg groups compared to the control group. Additionally, the FPN co-administered with the L-Arg group revealed a statistical upregulation in the mRNA of NOS compared to the FPN group.

## 4. Discussion

The existing study elucidates the effects of TT and L-Arg on various physiological and biochemical parameters in male Wistar rats under conditions of FPN-induced toxicity. The comprehensive analysis of body weight dynamics and reproductive health indicators revealed the significant potentials of TT and L-Arg, highlighting their antioxidative and endocrine-modulating properties. The identified metabolites from the methanolic extract of TT exhibited a diverse range of bioactive ingredients, including flavonoids, flavanones, anthocyanidin-3-O-glycosides, and hydroxycinnamic acids, as determined through LC-MS/MS analysis. These ingredients are well-documented for their antioxidative properties, which can overwhelm oxidative stress, a known contributor to reproductive toxicity and dysfunction [47]. When compared to other plant extracts known for similar bioactivities, TT stands out due to its unique combination of metabolites and their specific glycosylation patterns and the variety of chemical classes, such as glucosinolates and lignans, which are not commonly found in other popular medicinal plants, and enhance TT’s functional potential [48]. These antioxidants likely underpin the observed improvements in body weight and reproductive parameters in TT-treated groups, as free radicals can adversely affect sperm quality and overall male fertility [49].

The FPN-treated group exhibited the lowest sex organ weights, indicating the detrimental impact of FPN on reproductive health, which can be associated with endocrine disruption [7]. Notably, the significant increases in FBW and WG observed in the FPN + TT and FPN + L-Arg groups compared to the FPN groups suggest that these treatments counteract the weight-decreasing effects of FPN exposure. The enhanced relative weights of epididymis in these groups further underscore the potential of TT and L-Arg in promoting reproductive organ health.

Testosterone is crucial for the enhancement of sexual behavior and regulation of spermatogenesis [50]. The FPN group exhibited markedly low testosterone levels and poor sperm parameters, affirming the toxicological risks posed by FPN. The restoration of testosterone levels and improvement in sperm parameters in the FPN + TT and FPN + L-Arg groups suggest that both treatments can effectively ameliorate FPN-induced reproductive impairments. The significant improvement in sperm motility and vitality in these groups aligns with the findings of a previous study, indicating that both TT and L-Arg can enhance reproductive outcomes through testosterone modulation [51].

The behavioral assays demonstrated significant differences in sexual behavior parameters. FPN + TT- and FPN + L-Arg-treated rats exhibited prolonged mating behaviors. This behavioral enhancement is likely mediated by the increased testosterone levels and improved sperm quality observed in these groups. The findings indicate that both TT and L-Arg not only positively influence physiological and biochemical outcomes but also enhance sexual motivation and performance.

Fipronil’s detrimental effects on SOD and TAC emphasize the compound’s role in inducing oxidative damage, which is counteracted by the antioxidant properties of TT and L-Arg. Treated FPN with TT or L-Arg increased SOD and TAC, which points to the enhanced antioxidative defense mechanisms. The significant catalase activity and MDA content reductions in the treated groups corroborate these findings. The administration of TT and L-Arg appears to confer protective effects against FPN-induced histological damage in testicular and penile tissues. By counteracting oxidative stress and inflammation, these compounds not only preserve tissue integrity but may also enhance reproductive health outcomes.

Fipronil interfered with the normal functioning of AR, potentially leading to reduced responsiveness to androgens, which are critical for male reproductive health. Altered AR signaling can impact various physiological processes, including spermatogenesis, libido, and erectile function. FPN exposure may also influence the activity and the expression of NOS, the enzyme responsible for constructing NO, a vital mediator of vasodilation and blood flow in erectile tissues. Reduced NOS activity can lead to impaired erection and decreased blood flow, further compromising reproductive health. Treatment with L-Arg, an essential amino acid that serves as a precursor for NO synthesis [52], can boost NO production through enhanced NOS activity, which is decisive for upholding erectile function and vascular health [53]. L-Arg may also indirectly support AR activity by improving blood flow and enhancing the delivery of androgens to target tissues, thereby promoting typical androgen signaling [54]. L-Arg antioxidant properties can help mitigate oxidative stress associated with FPN exposure, preserving NOS activity and AR function. TT enhances testosterone levels, positively influencing AR expression and activity. Increased androgen levels can help counteract the inhibitory effects of FPN on AR function. The antioxidant compounds present in TT may reduce oxidative stress and inflammation, thereby supporting both NOS activity and AR signaling pathways [55]. These compounds are crucial for maintaining tissue integrity and function in the presence of FPN.

This study provides a detailed analysis of the effects of FPN and various treatments on histopathological and spermatogenic parameters. Using the Kruskal–Wallis test and Spearman’s rank-order correlation coefficient, this study revealed significant differences across the treatment groups, particularly concerning reproductive health markers. One of the most notable findings is the detrimental effect of FPN, as seen in the significant alterations in histopathological and spermatogenic parameters. The Masson’s trichrome staining of the penis showed a substantial reduction in collagen deposition in the FPN-treated group, indicating that FPN disrupts the structural integrity of penile tissue, which could impact male reproductive function [56]. The Johnson score, seminiferous tubule diameter, and spermatogenic height analysis further highlighted the negative impact of FPN, with significantly lower scores and smaller diameters observed compared to other treatment groups. This suggests that FPN interferes with spermatogenesis at multiple levels, from cellular proliferation to the overall architecture of the seminiferous tubules [9]. The observed reduction in spermatogenic height indicates impaired cellular development within the testes, consistent with existing evidence of fipronil’s toxicity to male fertility [57].

In contrast, the treatments involving TT and L-Arg showed protective effects. Both substances positively correlated with key spermatogenic indices, such as Johnson score, seminiferous tubule diameter, and spermatogenic height. These findings support previous studies indicating that TT and L-Arg have antioxidant and fertility-enhancing properties [58]. The significant positive correlations observed for these treatments suggest that they may help mitigate some of fipronil’s harmful effects, positioning them as potential therapeutic agents for improving male reproductive health under toxic stress.

An intriguing aspect of the study is the differential effects observed in Masson’s trichrome staining of the testes. While FPN treatment did not significantly impact collagen deposition in the testes, indicating a tissue-specific response, treatments with TT and L-Arg did not substantially alter testicular collagen levels. This suggests that the protective effects of these treatments are likely related to their influence on spermatogenic function and the structural integrity of seminiferous tubules rather than directly affecting collagen synthesis in the testes.

Spearman’s rank-order correlations further emphasize the interdependence of the measured parameters, with strong negative correlations between FPN and key spermatogenic indices, reinforcing its toxic effects on male fertility. On the other hand, the positive correlations between TT, L-Arg, and spermatogenic parameters suggest that these compounds support the maintenance of spermatogenesis and overall reproductive health. These findings are also supported by negative correlations between FPN and both TT and L-Arg, suggesting that the toxic effects of FPN can be counteracted by these treatments, particularly in enhancing spermatogenesis.

Fipronil exposure has been demonstrated to harmfully influence male reproductive health, potentially leading to disruptions in spermatogenesis. The reduction in PCNA expression in the testes of FPN-intoxicated rats indicates a decrease in the proliferation of germ cells, which is critical for the maintenance of sperm production. The decline in PCNA expression could result from oxidative stress induced by FPN, which may cause central DNA damage and subsequent cellular apoptosis in testicular tissue [59]. This impairment in germ cell proliferation can manifest as reduced sperm counts, decreased motility, and overall fertility challenges. In this study, the administration of TT and L-Arg showed marked statistical improvements in the testicular expression of PCNA compared with the FPN group. The enhanced expression of PCNA in these treatment groups suggests that both TT and L-Arg may promote cell proliferation and mitigate the toxic effects of FPN on testicular tissue. TT, as found for its antioxidant compounds, may reduce oxidative stress and protect germ cells from damage induced by FPN. By enhancing the antioxidant defense system, TT can promote a more favorable environment for cell proliferation [60]. As a precursor to NO, L-Arg can enhance blood flow and improve oxygen delivery to testicular tissues [61]. Increased NO levels can facilitate cellular signaling pathways that promote cell growth and regeneration [62]. Histopathological analyses corroborate the immunohistochemical findings. The restoration of PCNA expression in FPN + TT- and FPN + L-Arg-treated groups aligns with the improved histological architecture of the testes, including healthier seminiferous tubules and increased spermatogenic activity. This supports the notion that enhancing cellular proliferation through targeted therapies can counteract the damaging effects of FPN.

The mean rank analysis of PCNA expression further supported these findings, with the FPN group consistently showing the lowest mean ranks, indicating a significant detrimental effect compared to the other treatments. Both TT and L-Arg treatments, and their combinations with FPN, resulted in higher mean ranks of PCNA expression, which signifies their potential to reduce the severity of fipronil’s negative impact. The mean rank comparisons reinforced these results, with FPN treatment showing significantly lower mean ranks compared to L-Arg, TT, and their combinations, highlighting the toxic effect of FPN and the partial efficacy of TT and L-Arg in reducing these adverse consequences. These results suggest that while the herbal treatments and L-Arg have a mitigating effect, they fully counterbalance the toxic effects of FPN.

Spearman’s correlation analysis further revealed significant relationships between FPN exposure and PCNA expression, with FPN showing a negative correlation, suggesting that FPN may inhibit cellular proliferation in testicular tissues. The positive correlation between L-Arg and PCNA expressions suggests a potential activation effect on cellular proliferation, which could reflect its role in modulating oxidative stress or reducing apoptosis in damaged cells. On the other hand, TT showed a positive correlation with PCNA, supporting the notion that TT may stimulate cell proliferation to restore spermatogenesis. The combined treatments of FPN + TT showed a positive correlation, implying that TT may increase the proliferative effects of the spermatogenic cells, further suggesting its potential to mitigate FPN-induced testicular damage by regulating cellular proliferation.

## 5. Conclusions

This study’s findings indicate that TT and L-Arg significantly protect against FPN-induced reproductive toxicity in male rats. The enhancement of antioxidant defenses, restoration of testosterone levels, improvement in semen quality, and sexual behavior collectively point to the potential of these compounds in managing oxidative stress-related reproductive disorders in male Wistar rats. Ultimately, this study paves the way for future interventions aimed at mitigating the reproductive health risks associated with pesticide exposure and offers hope for developing effective, natural solutions to protect male reproductive health from environmental stressors. A salient limitation of the present study is the absence of direct investigation into the combined effects of L-Arg and TT extract. While individual administration of both compounds demonstrated protective properties, the potential for synergistic interaction remains speculative and necessitates further experimental validation to ascertain the nature of their combined effect. Furthermore, our reliance solely on immunohistochemistry without complementary quantitative protein validation techniques such as Western blotting constitutes a methodological constraint. This limitation restricts our ability to precisely quantify expression levels of key proteins involved in the protective mechanisms. Additionally, while our study demonstrates significant protective effects in an acute exposure model, the long-term efficacy of these interventions against chronic pesticide exposure remains to be elucidated. Future studies incorporating more extended observation periods would provide valuable insights into the sustained protective capacity of these compounds against persistent environmental toxicants and their potential application in clinical settings.

## Figures and Tables

**Table 1:** Primers of rat androgen receptor (AR), nitric oxide synthase (NOS), and β-actin genes for real-time PCR.

Gene	Sequence (5′–3′)	Annealing Temperature	Amplicon Size (bp)	Accession Number
AR [45]	F: 5′-AATGTACAGCCAGTGCGTGA-3′	57	248	NM_012502.2
	R: 5′-TTGGTGAGCTGGTAGAAGCG-3′			
NOS [46]	F: 5′-CATTGGAAGTGAAGCGTTTCG-3′	58	95	L12562
	R: 5′-CAGCTGGGCTGTACAAACCTT-3′			
β-actin [46]	F: 5′-AAGTCCCTCACCCTCCCAAAAG-3′	60	98	V01217
	R: 5′-AAGCAATGCTGTCACCTTCCC-3′			

## Data Availability

The original contributions presented in this study are included in the article/Supplementary Materials. Further inquiries can be directed to the corresponding authors.

## Supplementary Materials

The following supporting information can be downloaded at: https://www.mdpi.com/article/10.3390/toxics13050371/s1, Table S1: Identified compounds of *Tribulus terrestris* (TT) found in both negative and positive modes of LC-MS/MS analysis.

## Abbreviations

TT	*Tribulus terrestris*
L-Arg	L-Arginine
FPN	Fipronil
AR	Androgen Receptor
NOS	Nitric Oxide Synthase
FBW	Final Body Weight
WG	Weight Gain
SOD	Superoxide Dismutase
TAC	Total Antioxidant Capacity
MDA	Malondialdehyde
PCNA	Proliferating Cell Nuclear Antigen
qRT-PCR	Quantitative Real-Time Polymerase Chain Reaction
H&E	Hematoxylin and Eosin
LH	Luteinizing Hormone
NO	Nitric Oxide
