# Supplementary material for: Modulating Effects of L-Arginine and Tribulus terrestris Extract on Fipronil-Induced Interference in the Male Reproductive System of Rats: Antioxidant Potential, Androgen Receptors, and Nitric Oxide Synthase Interplay
Source: Toxics. 2025 May 2;13(5):371. doi: 10.3390/toxics13050371 (PMC12115952; doi:10.3390/toxics13050371)
Supplement: Supplementary file 1 [file toxics-13-00371-s001.zip › toxics-3573765-supplementary.pdf]

Supplement Table S1: Identified compounds of *Tribulus terrestris* (TT) found in both negative and positive modes of LC-MS/MS Analysis

	Title	RT (min)	Experimental Precursor *m/z*	Reference Precursor m/z	PPM shift	MSMS spectrum	Adduct	Formula	Ontology
1	Glucosinalbin 4-(4-acetylrhamnoside)	3.13	612.105585	612.10624	-1.1	96.9608 [HO4S]-, 259.0131 [C8H7NO5S2-H]-H-, 274.9902 [C15H18O5-2H]-H-, 370.0964 [C16H19NO7S+H]-	$[M-H]^-$	$C_{22}H_{31}NO_{15}S_2$	Alkylglucosinolates
2	Uridine diphosphate acetylgalactosamine 4-sulfate	3.13	686.03764	686.03111	9.5	96.9604 [H2O4P]-, 274.9978 [C5H10O9P2]-H-, 612.0953 [C14H21N3O18P2S]-H-	$[M-H]^-$	$C_{17}H_{27}N_3O_{20}P_2S$	Pyrimidine ribonucleoside diphosphates
3	p-Coumaroyl vitisin A	3.34	706.13855	706.15393	-21.8	106.9621 [C7H6O+H]-, 166.0329 [C9H9O3+H]-, 227.0061 [C12H5O5-H]-H-, 310.0572 [C15H17O7+H]-, 506.1154 [C26H21O11-2H]-H-	$[M-H]^-$	$C_{35}H_{31}O_{16}$	Hydroxycinnamic acids and derivatives
4	Kelampayoside A	3.72	477.16009	477.16137	-2.7	123.045 [C7H8O2]-H-, 145.05 [C6H10O4]-H-, 307.104 [C12H18O9+H]-, 431.159 [C18H24O12]-H-	$[M-H]^-$	$C_{20}H_{30}O_{13}$	Phenolic glycosides
5	beta-Glucogallin	3.79	331.0671398	331.06707	0.2	160.9218 [C5H7O6-H]-H-, 178.9311 [C8H5O5-H]-H-, 188.9422 [C7H10O6]-H-,	$[M-H]^-$	$C_{13}H_{16}O_{10}$	Tannins

						313.0710 [C13H15O9-H]-H-			
6	D-Vacciniin	5.33	283.0823435	283.08233	0	92.9273 [C5H3O2-H]-H-, 102.9718 [C7H5O-H]-H-, 112.9858 [C5H5O3]-, 130.9658 [C5H8O4]-H-, 146.9604 [C5H9O5-H]-H-	$[M-H]^-$	$C_{13}H_{16}O_7$	Benzoic acid esters
7	Naringenin 5-O-glucuronide or naringenin-7-O-glucuronide	5.49	447.09202	447.09329	-2.8	102.9589, 112.9845,130.9635 [C5H8O4]-H-, 174.9557 [C6H9O6-H]-H-, 226.9343 [C13H9O4-H]-H-, 327.0554 [C13H12O10]-H-, 357.0602 [C18H14O8]-H-, 401.1454 401.1454, [C20H19O9-H]-H-, 429.0734	$[M-H]^-$	$C_{21}H_{20}O_{11}$	Flavonoid-7-O-glucuronides
8	Sambunigrin	5.56	294.0980408	294.09831	-0.9	76.9732 [C6H5]-, 104.9539 [C7H6O]-H-, 114.9878 [C8H6N]-H-, 132.9286 [C8H7O2-H]-H-, 148.0405 [C5H8O5]-	$[M-H]^-$	$C_{14}H_{17}NO_6$	Cyanogenic glycosides
9	Rutin (Quercetin 3-rutinoside)	6.00	609.1460722	609.14611	5.97905	151.0058 [C7H4O4]-H-, 271.0243 [C14H9O6-H]-H-, 300.0269 [C15H9O7]-H-, 343.0372 [C17H12O8]-H-	$[M-H]^-$	$C_{27}H_{30}O_{16}$	Flavonoid-3-O-glycosides

10	Spiraeoside (Quercetin 4'-O-glucoside)	6.48	463.0863506	463.0882	0.2	151.0013 [C7H4O4]-H- , 174.9552 [C9H4O4]-H-, 242.931 [C13H8O5]-H-, 271.0231 [C12H15O7]-, 300.0261, 301.0353 [C15H9O7]-	$[M-H]^-$	$C_{21}H_{20}O_{12}$	Flavonoid O-glycosides
11	Gingerol	9.40	293.17632	293.17583	1.7	102.9562 [C7H6O-2H]-H-, 112.9863 [C6H10O2]-H-, 148.0542 [C9H8O2]-, 177.0924 [C10H11O3-H]-H-, 192.1169 [C11H13O3]-H-, 205.1235 [C12H14O3]-H-, 221.1547 [C12H15O4-H]-H-	$[M-H]^-$	$C_{17}H_{26}O_4$	Gingerols
12	Dehydrophytosphingosine	14.38	316.2847	316.2846	0.3	60.0443, 95.0881, 225.08981, 280.2640, 298.8992	$[M+H]^+$	$C_{18}H_{37}NO_3$	Amines
13	Phytosphingosine	13.21	318.3001	318.2994	2.2	57.699 [C4H9]+, 70.0654, 88.0750 [C5H10O+H]+H+, 219.1740 [C11H24NO3]+H+, 300.2903 [C18H38NO2]+	$[M+H]^+$	$C_{18}H_{39}NO_3$	Amines
14	Spermidine	1.06	146.16489	146.16517	-1.9	68.9825 [C4H9N-2H]+, 72.0805 [C4H10N]+, 84.0792 [C5H11N-H]+, 112.1137 [C7H15N-H]+, 129.1389 [C7H17N2]+	$[M+H]^+$	$C_7H_{19}N_3$	Dialkylamines

15	L-Histidine trimethylbetaine	1.10	198.123027	198.1237	-3.4	60.0810 [C3H9N]+H+, 68.0495 [C4H5N]+H+, 95.0595 [C5H6N2]+H+	$[M+H]^+$	$C_9H_{15}N_3O_2$	Histidine and derivatives
16	Valyl-Lysine	1.10	246.1802215	246.18122	-4.1	58.0669 [C3H8N]+, 100.0754 [C5H10NO]+, 117.1023 [C5H11N2O+H]+H+	$[M+H]^+$	$C_{11}H_{23}N_3O_3$	Dipeptides
17	Occidentoside	1.16	705.1843	705.1814	4.1	423.0801 [C22H14O9]+H+, 525.1356 [C27H25O11]+, 543.131 [C30H21O10+H]+H+	$[M+H]^+$	$C_{36}H_{32}O_{15}$	Lignan glycosides
18	Yuccaol C or Rugulosin	1.17	543.1308	543.12857	4.1	122.9259 [C6H4O3-H]+, 381.0757 [C21H14O7+2H]+H+	[M+H]+	$C_{30}H_{22}O_{10}$	2-arylbenzofuran flavonoids
19	Trisjuglone	1.21	517.0504158	517.05541	-9.7	350.1012 [C22H7O5-H]+, 365.1012 [C22H7O6-2H]+, 381.0766 [C23H7O6+H]+H+, 471.0323 [C29H11O7]+	[M+H]+	$C_{30}H_{12}O_9$	Triphenylenes
20	Glucobrassicin	1.21	449.0646664	449.0683	-8.1	203.0548 [C10H8N2OS-H]+, 226.9774 [C4H6NO6S2-H]+, 283.0073 [C11H9N2O3S2+H]+H+, 365.1061 [C11H13N2O8S2]+, 381.0811 [C12H15N2O8S2+H]+H+,	[M+H]+	$C_{16}H_{20}N_2O_9S_2$	Alkylglucosinolates

						403.0577 [C14H16N2O8S2-H]+			
21	Phlorin or Glucosylisomaltol	1.23	289.0903	289.09179	-5.2	127.0428 [C6H5O3+H]+H+	[M+H]+	$C_{12}H_{16}O_8$	Phenolic glycosides
22	Gerberinol	1.28	365.1048	365.10196	7.8	127.0383 [C6H4O3+2H]+H+, 185.0415 [C12H8O2]+H+, 203.0519 [C12H9O3+H]+H+	[M+H]+	$C_{21}H_{1}6O_6$	4-hydroxycoumarins
23	Phenylalanine	2.19	166.0863	166.08626	0.2	71.9514 [C3H4O2]+, 77.0381 [C6H5]+, 84.9595 [C4H4O2]+H+, 103.0537 [C8H8-H]+, 120.081 [C8H10N]+	[M+H]+	$C_9H_{11}NO_2$	Phenylalanine and derivatives
24	2,6-Di-tert-butyl-4-ethylphenol	3.09	235.20516	235.20564	-2	95.0861 [C7H10]+H+, 159.0658 [C12H16-H]+, 217.1948 [C16H25]+	[M+H]+	$C_{16}H_{26}O$	Phenylpropanes
25	5-O-beta-D-Xylopyranosyl-L-arabinose or 5-O-a-L-Arabinofuranosyl-L-arabinose	3.11	283.102529	283.10236	0.6	85.0273 [C4H7O2-2H]+, 120.0470 [C4H7O4]+H+, 122.0442 [C4H8O4+H]+H+	[M+H]+	$C_{10}H_{18}O_9$	glycoside

26	1-(2-Hydroxyphenylamino)-1-deoxy-beta-D-gentiobioside 1,2-carbamate	3.13	460.14131	460.14495	-7.9	184.0426 [C7H7NO5-H]+, 298.0823 [C13H14NO7+H]+H+, 346.1012 [C13H17NO10-H]+	[M+H]+	$C_{19}H_{25}NO_{12}$	glycoside
27	Sceptrin	3.13	619.0486506	619.05232	-5.9	142.9397 [C4H3BrN-H]+, 336.1073 [C13H14BrN5O]+H+, 352.0869 [C17H20N8O]+, 457.1026 [C19H20BrN7O2]+, 514.1395 [C20H23BrN10O2]+	[M+H]+	$C_{22}H_{24}Br_2N_{10}O_2$	
28	Monoiodothyronine	3.14	400.00544	400.00404	3.5	158.0997 [C10H7O2-H]+, 184.0265 [C12H8O2]+, 304.0835 [C10H8INO2+2H]+H+, 314.956 [C11H8IO3]+, 320.0548 [C10H9INO3+H]+H+	[M+H]+	$C_{15}H_{14}INO_4$	Phenylalanine and derivatives
29	Vaccinoside	3.14	537.15935	537.16027	-1.7	184.0539 [C9H9O4+2H]+H+, 282.0521 [C13H12O7+H]+H+, 416.0628 [C17H21O12-H]+, 520.1366 [C25H27O12]+H+	[M+H]+	$C_{25}H_{28}O_{13}$	Iridoid O-glycosides

30	Cepharadione A	3.18	306.0764812	306.07608	1.3	146.0336 [C8H3O3-H]+, 162.0167 [C9H6NO2+H]+H+, 185.0433 [C11H8NO2-H]+, 204.0289 [C15H9N]+H+, 228.0303 [C12H7NO4-H]+, 258.0426 [C17H9NO2-H]+	[M+H]+	$C_{18}H_{11}NO_4$	Aporphines
31	Indoleacrylic acid	3.21	188.0692855	188.07061	-7	84.9599 [C4H3O2+H]+H+, 91.0537 [C6H5N]+, 118.0653 [C8H6N+H]+H+, 143.0735, 146.0594 [C9H6NO+H]+H+, 170.057 [C11H8NO]+	[M+H]+	$C_{11}H_9NO_2$	Indoles
